# Evidence on Human Exposure to Pesticides and the Occurrence of Health Hazards in the Brazilian Population: A Systematic Review

**DOI:** 10.3389/fpubh.2021.787438

**Published:** 2022-01-07

**Authors:** Carolina Panis, Aedra Carla Bufalo Kawassaki, Ana Paula Jaqueline Crestani, Claudiceia Risso Pascotto, Durcelina Schiavoni Bortoloti, Geraldo Emílio Vicentini, Léia Carolina Lucio, Mariane Okamoto Ferreira, Rosebel Trindade Cunha Prates, Valquíria Kulig Vieira, Shaiane Carla Gaboardi, Luciano Zanetti Pessoa Candiotto

**Affiliations:** ^1^Grupo de Estudos Avançados em Ciências da Saúde (GEACS), Centro de Ciências da Saúde, Universidade Estadual Do Oeste Do Paraná, Francisco Beltrão, Brazil; ^2^Programa de Pós-graduação em Ciências Aplicadas à Saúde, Centro de Ciências da Saúde, Universidade Estadual Do Oeste Do Paraná, Francisco Beltrão, Brazil; ^3^Programa de Pós-Graduação em Geografia, Universidade Estadual Do Oeste Do Paraná (Unioeste), Francisco Beltrão, Brazil

**Keywords:** maternal exposure/adverse effects, pesticides exposure/adverse effects, occupational exposure/adverse effects, risk factors, congenital abnormalities

## Abstract

Brazil is among the biggest pesticide consumers in the world, with its population severely exposed to tons of such substances, both because of environmental contamination and occupational use. The health consequences of pesticide exposure are well-documented, but still sparse regarding Brazilian population. This study systematically reviewed the Brazilian studies published that address the relationship between exposure to pesticides and health problems in the Brazilian population. Also, information about pesticide use in Brazil is provided. The included studies showed that exposure to pesticides has a relevant impact on the health of the Brazilian population, regardless of age and gender, and on workers in rural areas or not. Most poisoning events seem to result from the continuous use of pesticides, whether occupationally or environmentally, characterizing a public health problem. The major consequences reported in literature were damage to the central nervous system, cancer, deleterious effects on rural workers' health, intoxications, malformations, and endocrine changes. These findings point out the need to understand the impact of chronic exposure to pesticides on severely exposed people and highlight the importance of creating public policies to protect them and avoid disease occurrence.

## Introduction

Pesticides are chemical and natural products of wide use commonly applied in agriculture to control or eliminate all types of pests, whether represented by insects, birds, or microorganisms that cause diseases in plants and weeds (1, 2). The use of pesticides is not restricted to agriculture: they are commonly used to control domestic pests, disease vectors, and home gardens. All pesticides are toxic by nature and pose serious risks to human health and the environment, mainly when used extensively and without safety measures (3).

One of the biggest pesticide consumers in the world is Brazil. Thus, the population is exposed to pesticides both because of environmental contamination and intentional use (4). Brazil is one of the biggest consumers of pesticides in the world (5, 6) and 80% of the pesticides authorized in the country are not allowed in at least three countries of the Organization for Economic Cooperation and Development (OECD) of the European community (7). Still, the list of active ingredients present in pesticides authorized in Brazil includes some of known toxicity to human health and the environment (7). It is estimated that each Brazilian consumes an average of seven liters of pesticides per year, as it is directly related to the 70,000 acute and chronic poisonings in our country, according to data from the dossier prepared by the Brazilian Association of Public Health (ABRASCO) (8). The Brazilian Ministry of Health warns that, for each notified pesticide poisoning event, there are another 50 not reported (9). It is known that the most affected, regarding unintentional poisoning in Brazil, are, proportionally, children (10). The national statistics of poisoning in children shows a predominance (58.5%) of the accidental cause, followed by suicide attempts by adolescents. For every three accidental cases, there is an attempt or a suicide by young people (11).

The impact of human exposure to pesticides has been widely debated by the international scientific community. The International Cancer Research Agency (12) has gathered evidences on the potential of pesticides to cause cancer, and warns that some pesticides classified as persistent organic pollutants (such as DDT, lindane, and aldrin-dieldrin) and others currently in use (such as alachlor, glyphosate, and diuron) pose a threat to the exposed population, since they are classified as probably or possibly carcinogens. Pesticide exposure can be occupational or occasional, and occur through ingestion, inhalation, or contact with the skin and are generally related to the handling of these products (13). It is widely believed that exposure to pesticides is linked to various health disorders, such as respiratory and reproductive disorders, endocrine disruption, Hodgkin and non-Hodgkin lymphomas, Parkinson's disease, and various types of tumors such as breast, brain and prostate, lung, liver, lymphoid tissue, uterus, and thyroid (14). In Brazil, most of them have been linked to acute and chronic intoxications due to repetitive exposure to very toxic substances, as organophosphates and carbamates (15). It reinforces the need to understand the picture of pesticides and health problems in Brazil.

Considering the very sparse literature about exposure to pesticides and health problems, it is necessary to gather the published studies on the exposure of the Brazilian population to understand the contamination profile, where it has been reported with higher frequency, and which injuries have been attributed in this context. Literature shows that countries that are part of the top five pesticide consumers in the world, such as China and the United States, have published numerous studies on the impacts of these substances on the health of their population. Meanwhile, as far as we know, there are few studies with this focus on the Brazilian population. If we also consider the growing permissiveness that the country has been showing for the registration of pesticides, added to the fact that there is no effective legislation that even protects the population against this chronic and continued exposure, the justification for the need to go deeper into the chosen topic is enormous. In this context, this paper reviewed the main studies published concerning the occupational or occasional exposure of the Brazilian population to pesticides, addressing the main findings regarding the health problems related to this situation reported in the literature. Also, data about the Brazilian landscape considering its position as one of the main pesticide consumers in the globe are presented.

## Methods

Data from the official government agencies about high pesticide trade in Brazil are discussed. The systematic review of studies regarding human exposure was based on the PRISMA 2020 method guidelines. The PICO (16) framework (Patient/Problem or Population; Intervention or Investigated condition; Comparison condition; Outcome) was applied. The review aimed to find scientific studies in the databases that addressed possible relationship between exposure to pesticides and health problems in the Brazilian population.

### Eligibility Criteria

All the works that addressed the topic of pesticides and health implications/problems; that had primary data collected in Brazil; that were in the languages: Portuguese, Spanish, or English, and that had full text availability were included. Exclusion criteria were articles that partially addressed the topic; preprints; studies in the form of theses, dissertations, videos, or books; letters to the editor; that showed results from experimental studies in laboratory animals; and duplicate articles.

### Sources of Information, Research, and Study Selection

Searches were conducted in the electronic databases PubMed (https://pubmed.ncbi.nlm.nih.gov/) and Scientific Electronic Library Online (SciELO). In the PubMed database, the terms “Brazil pesticides” were used, with the search filters: free full text and humans (species), and filters by types of articles (article types: case report, classical article, clinical study, journal article, multicenter study, newspaper article, observational study, validation studies). In the SciELO database, the term: “pesticide” was used, filters: Brazil; type of literature: article, case report, and brief report. This search included papers published in the period from 1971 to July 20th 2021.

**Figure 1:**
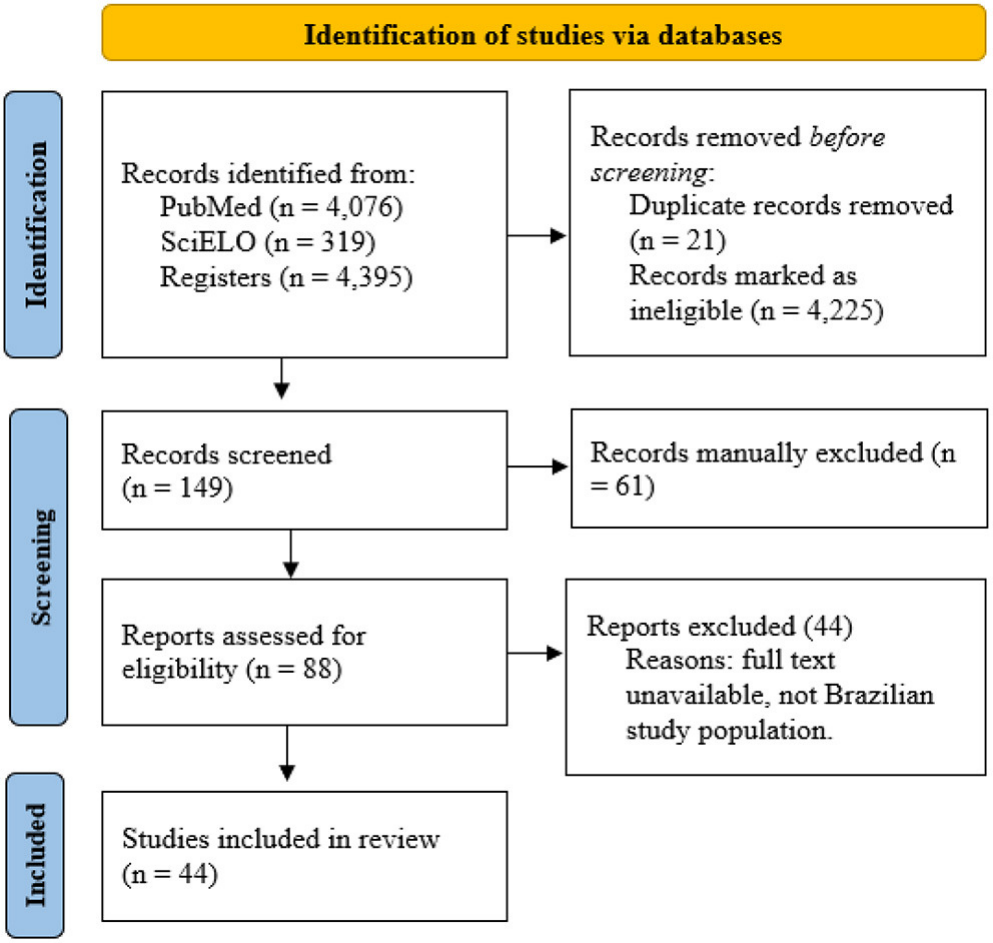
PRISMA 2020 flow diagram for updated systematic reviews, which included the search in databases, registers, and other sources.

In total, this search found 4,076 published works, of which 88 were selected according to the aforementioned eligibility criteria, as detailed in Figure 1. Five evaluators carried out the selection process of studies for review and those underwent a screening in two phases: initially, the studies were sorted by titles and abstracts and, if they were considered potentially relevant, these were moved to a later stage in which the full articles were selected for data extraction. All evaluators conducted the screening of records for study eligibility independently and with no automation tool. After selecting the articles for the study (44 articles), the classification/separation of the articles by health problem categories was carried out. There was no gray literature search (Figure 1).

### Data Collection and Extraction Process

The harm categories were classified as: worker's health; poisoning; malformations; endocrine disruptions; neoplasms; neurological changes; and other types of injury. Through categorization, information was extracted from the articles about: research location; population category (men, women, and children); population size; type of exposure to pesticides (occupational, environmental, direct, indirect, intoxication-ingestion, and vertical); outcome to the health of interest; conclusion; and the work methodology. The data of interest for the selected articles were collected by the authors themselves according to the division of themes. Statistical data, when available, were presented as mentioned in the original article.

Figure 1 shows the data collected. Given the heterogeneity of the categories of the found studies (types of studies, population of interest, region, and type of exposure), statistical studies were not used to explore the found results. Data from all included studies is available in Table 1.

**Table 1:** Detailed data of the included studies.

**References**	**City, state**	**Population**	**Type of exposure**	**Pesticides reported**	**Health hazard outcome**	**Study approach**	**Main findings**	**Thematic axis**
Santos Filho et al. (17)	Cubatão, São Paulo	251 children, from 1 to 10 years old	Food contamination. Consumption of fish and other products from the rivers of Cubatão.	Organochlorines HCB, HCH, p,p'DDE, o,p'DDT, and p,p'DDT.	Pesticide contamination	The study compared large fish-consumer children and non-consumer children.	73 children (30%) had p,p'DDE concentrations (mean = 0.85 ± 2.13 μg/l.), and in 47 children (19 %) total HCH was found (mean = 0.28 ± 0.79 μg/l.).	Intoxication and others
Koifman et al. (18)	Bom Jesus do Tocantins, Pará	44 Indigenous, 20–29 years old	Environmental	DDT and organophosphorus pesticides	Cancer risk	Analysis of blood and hair for pesticide detection	High blood levels of p,p'-DDT were observed and low exposure to organophosphorus pesticides.	Cancer
Mendonça et al. (19)	Rio de Janeiro, Rio de Janeiro	177 women	Environmental/ Accidental	DDE 1,1-Dichloro-2, 2-bis(p-chlorophenyl)ethylene	Cancer risk	HPLC-based pesticide dosage and exposure history (interview)	The study reported no risk.	Cancer
Araújo et al. (20)	Vale do São Francisco and Camocim de São Félix, Pernambuco	186 rural workers	Intoxication, occupational, and direct or indirect ingestion	Pesticides in general	Symptoms of disease	Interview	Dizziness, nausea, and headache were reported. 70.6% of women reported the loss of a fetus and 39.4% lost a child <1-year old. Problems related to the immune system had a greater number of complaints (36.4%): frequent fever and itchy skin, eyes, and nose; the most frequent symptom related to the musculoskeletal system was pain in the joints (35.8%), while the central and peripheral nervous systems were responsible for 32.5% of the complaints, with dizziness, tingling in the upper limbs being the most cited.	Worker's health and poisoning
Delgado et al. (21)	Rio de Janeiro, Rio de Janeiro	33 volunteers (16 men and 17 women)	Environmental	Organochlorine residues (o,p'DDT, p,p'DDT, p,p'DDD, p,p'DDE, Aldrin, Dieldrin, Endrin, Heptachlor, Heptachlor-epoxide, α-, β- and γ-Hexachlorocyclohexane, Hexachlorobenzene).	Contamination study	Measurement of pesticide residues in peripheral blood samples.	P,p'DDE was found in 17 of the 33 samples at concentrations ranging from 1.4 to 8.4 microg/L of serum or, on a fat basis, from 0.200 to 3,452 microg/g of serum lipids. Percentage of positive samples (%) and p,p'DDE levels (range of positive samples) increased from the youngest to the oldest group.	Poisoning and endocrine disruption
Fonseca et al. (22)	Curitiba, Paraná	134 boys and girls	Environmental	Pesticides in general	Occurrence of severe aplastic anemia	Interview	Agricultural pesticides and benzene derivatives were the most reported possible causal factors (42%)	Others
Koifman et al. (23)	People from 11 Brazilian states	Population from the 11 states (not clear in the study)	Environmental	Insecticides, herbicides, fungicides.	Human reproductive disorders: mortality due to cancer in testicle, prostate, female breast or ovary, authorization of chemotherapy procedures, infertility (spermogram and hysterosalpingography analysis and testicular correction surgeries).	Database study	Pearson's correlation coefficients were verified between pesticide sales data available in eleven states in Brazil in 1985 and selected other reproductive outcomes or their substitutes. Moderate to high correlations were observed for infertility, testicle, death from breast, prostate, and ovarian cancer.	Endocrine disruption
Leite et al. (24)	Rio de Janeiro, RJ	822 Children	Environmental and parental exposures	Household insecticides and other chemicals	Congenital malformations	Secondary-based case-control study	Proximity to industrial installations as a risk factor for all orofacial clefts, as well as the combined use of household insecticides and urban vector control pesticide spraying. Domestic services was a group of maternal occupation heavily associated with orofacial clefts	Worker's health and malformations
Soares et al. (25)	Nine cities in Minas Gerais	1,064 adults and children over 10 years old	Intoxication and occupational exposure	Organophosphates and carbamate	Intoxication risk	Interview and blood analysis determine cholinesterase activity	Most of the interviewed people had direct contact with pesticides and were moderately intoxicated. Unprotected rural worker has up to 72% chance of becoming intoxicate.	Worker's health and poisoning
Delgado and Paumgartt (26)	Paty do Alferes, Rio de Janeiro	55 Adults	Intoxication and occupational exposure	Insecticides (abamectin, organophosphate compounds, and pyrethroids), and fungicides such as mancozeb, chlorothalonil, and copper-based products.	Occupational exposure profiles	Interview	92% did not use any personal protection equipment, 77% shower immediately after application and/or preparation, 62% have been sick while preparing or using pesticide.	Worker's health and poisoning
Faria et al. (27)	Two cities in Rio Grande do Sul	1,379 rural workers, over 15 years old	Occupational and environmental exposures	Pesticides in general	Respiratory symptoms	Interview	A positive linear association between exposure frequency and respiratory symptoms is reported, increase in risk from 70 to 90% among individuals who worked on more than one farm, prepared chemical mixtures, and washed contaminated clothes, when compared to those who did not perform these activities.	Worker's health, poisoning, and others
Peres et al. (28)	Nova Friburgo, Rio de Janeiro	650 individuals	Occupational exposure	Pesticides in general	Intoxication profiles	Interviews	The perception of workers about health risks involves episodes of acute intoxication leading to symptoms such as headaches, disorientation; convulsions; nausea; shortness of breath; and vomiting.	Worker's health, poisoning, and SNC
Pires et al. (29)	Dourados, Mato Grosso do Sul	475 intoxicated individuals	• Occupational and environmental exposures • Food contamination	Agricultural pesticides (especially insecticides—organophosphates and carbamates)	Intoxication, Mortality, and Suicide	Epidemiological research based on notification records	475 poisonings were from pesticide use, 261 were accidental or professional, 14 people died from intoxication. High prevalence of poisoning (64.5) and suicide attempts (58.7) per 100,000 inhabitants of the rural population. Significant correlation between intoxication and suicide attempts (*r* = 0.60; *p* < 0.05) and between suicide attempts and the proportion of the area occupied by temporary crops (*r* = 0.68; *p* < 0.05).	Worker's health and poisoning
Pires et al. (30)	Mato Grosso do Sul	1,355 intoxicated individuals	Intoxication, occupational exposure, and direct or indirect ingestion	Insecticides and herbicides used in agriculture	Intoxication, Mortality, and Suicide	Observational study based on intoxication reports.	Correlations between the prevalence of suicide attempts and the prevalence of deaths corresponding to culture.	Worker's health and poisoning
De Godoy Martins et al. (31)	Londrina, Paraná	473 Children under 15 years old in the year 2000	Intoxication, occupational and direct or indirect ingestion	Pesticides in general	Morbi-mortality from poisoning	Cross-sectional and descriptive study on morbidity and mortality from poisoning. Information obtained from medical records.	A higher risk of poisoning was observed between 1 and 3 years old, due to exposure to harmful substances, among which 14.1% were pesticides, the second most frequent.	Intoxication
Werneck et al. (32)	Rio de Janeiro, Rio de Janeiro	160 patients	Direct or indirect ingestion of pesticides and other drugs	Poisoning by “chumbinho,” a product illegally sold as rodenticide, which generally contains the pesticide carbamate in its composition.	Suicide attempts	Observational study based on intoxication reports	Ingestion of pesticides and prescription drugs were the two most common methods reported, and had been used by similar numbers of women, while two-thirds of men used pesticides.	Intoxication and others
Araújo et al. (33)	Nova Friburgo, Rio de Janeiro	102 individuals	Intoxication and occupational exposure	Pesticides in general	Intoxication, late neuropathy and neurobehavioral syndrome, and neuropsychiatric disorders.	Observational cross-sectional epidemiological study	12.8% of cases of late neuropathy and 29 (28.5%) cases of neurobehavioral syndrome and neuropsychiatric disorders were associated with the chronic use of pesticides	Worker's health, poisoning, and SNC
Meyer et al. (34)	Luz, Minas Gerais	50 individuals	Intoxication, occupational exposure, and direct or indirect ingestion	Organophosphates and carbamates.	Suicide attempts	Medical records and intoxication reports	Suicide attempts by pesticide ingestion was found in 57.9% of cases	Worker's health and poisoning + other
Fróes Asmus et al. (35)	Duque de Caxias, Rio de Janeiro	1,199 individuals	Environmental exposure and food contamination	Organochlorine HCH and its isomers (in eggs, milk, and soil); DDT and its metabolites; trichlorobenzenes; trichlorophenols; dioxins; and furans.	Cancer Risk	Analysis of contaminants in samples of topsoil and food (eggs and cow's milk).	Exposures to DDT and dioxin exceed the minimum acute, intermediate, and chronic risk levels in soil and eggs/milk. Excessive risk of cancer for children and adults due to exposure has also been found.	Intoxication and others
Ferreira et al. (15)	Paraná	529 individuals	Food contamination, intoxication, and occupational exposure	Organophosphate insecticides and carbamates	Intoxication	Intoxication reports	140 patients intoxicated due to occupational exposure to pesticides and 2 patients died. Ingestion of organophosphates and carbamates was much more serious than intoxication due to occupational or accidental exposure.	Worker's health, poisoning, and others
Presgrave et al. (36)	Rio de Janeiro, Rio de Janeiro	2,810 children	Intoxication	Pesticides in general	Accidental intoxication	Descriptive statistical analysis, using the Epi Info program.	Pesticides were among the main products ingested by children	Intoxication and others
Maluf et al. (37)	Cities of Manaus, Recife, Goiânia, Juiz de Fora, Uberaba, Ribeirão Preto, Curitiba	224 individuals	Environmental exposure	Herbicides	Risk assessment study for aplastic anemia occurrence	Interview	High rates of benzene exposure (≥30 exposures per year) were associated with a high risk of aplastic anemia (odds ratio, OR: 4.2; 95% confidence interval, CI: 1.82–9, 82). Individuals exposed to chloramphenicol in the previous year had an adjusted OR for aplastic anemia of 8.7 (CI: 0.87–87.93) and those exposed to azithromycin had an adjusted OR of 11.02 (CI 1.14–108).	Others
Werneck and Hasselmann (38)	Rio de Janeiro, Rio de Janeiro	1,574 cases of poisoning in children up to 5 years old	Intoxication	Pesticides in general	Intoxication	Emergency care bulletins	From 1,574 cases of poisoning detected, about 15% were caused by pesticide ingestion. More than half of these involved the rodenticide substance named chumbinho.	Intoxication
Silva et al. (39)	Petrolina, Pernambuco	42 cases and 84 controls	Environmental and occupational exposures	Pesticides in general	Diagnosis of newborns with congenital defects.	Risk assessment study	Increased risk of birth defects when the following were considered: both parents working on the farm and living nearby, maternal home close to the farm, parent working on the farm, parent applying the products on the farm, and exposure of at least one parent. In none of the situations, however, there was a significant difference.	Worker's health and malformations
Veras et al. (40)	Recife, Pernambuco	25 Female adolescents between 13 and 19 years old	Intoxication	Pesticides in general	Suicide attempts	Interviews and medical records	About 60% of the cases involved pesticide ingestion	Intoxication
Cremonese et al. (41)	States of Paraná, Santa Catarina, and Rio Grande do Sul	Parturients and newborns	Environmental	Pesticides in general	The prevalence of prematurity, low birth weight, and reduced Apgar score (<8) in the 1st and 5th min were determined	Ecological study	Apgar scores <8 was highly prevalent in the upper quartile of pesticide consumption, and no significance was detected for low birth weight.	Endocrine disruption and malformations
Bastos et al. (42)	Rio de Janeiro, Rio de Janeiro	36 women	Environmental	Organochlorine pesticides and polychlorinated biphenyls (PCB).	Female infertility	Medical records and blood analysis	p,p'DDE was detected in 100% of infertile women, with levels higher than in the pregnant ones (3.02 mcg/L vs. 0.88 mcg/L; *p* = 0.001; power 69%), with no correlation in the etiology of infertility. Only PCB180 showed significance in the frequency of 71.4% (*p* = 0.019).	Endocrine disruption
Curvo et al. (43)	State of Mato Grosso	702 children, 0–19 years old	Environmental	Glyphosate, endosulfan, 2.4 D, and tebuconazole.	Cancer morbidity and mortality	Database study	Average use of pesticides showed a statistically significant association for both morbidity (*p* = 0.021) and mortality (*p* = 0.005) from childhood cancer, with a 95% confidence interval.	Cancer
Ferreira et al. (44)	13 Brazilian states	252 mothers and 423 controls	Environmental	Pesticides from multiple chemical classes (for instance, pyrethroids, organophosphates, and carbamates), or maternal exposure to both insecticides and herbicides	Occurrence of leukemia in babies from exposed mothers	Interview	Associations with pesticide use during pregnancy were observed for acute lymphoid leukemia (ALL) (aOR = 2.10; 95% CI: 1.14, 3.86) and acute myeloid leukemia (AML) (aOR = 5.01; 95% CI: 1.97, 12.7) in children aged 0–11 months.	Cancer
Santos et al. (45)	Rio de Janeiro, Rio de Janeiro	940 cases	Environmental	Pesticides in general	Suicide or attempted suicide.	Database study	Females between 20–39 years old prevailed, as well as the use of medicines and pesticides.	Intoxication
Campos et al. (46)	Duque de Caxias, Rio de Janeiro	102 children aged 6–16 years	Environmental	Organochlorine pesticides (OC)	Cognitive development impairment	Interview and laboratory tests	Chronic pesticide exposure may affect the cognitive capacity of children	Endocrine disruption and SNC
Cremonese et al. (47)	States of Paraná, Rio Grande do Sul, Santa Catarina, Espírito Santo, Minas Gerais, Rio de Janeiro, and São Paulo.	29,925 individuals	Environmental	Pesticides in general	central nervous system and cardiovascular congenital malformations	Population-based Ecological Study	The findings suggest that prenatal exposures may be related with the occurrence of certain congenital defects	Malformations and SNC
Faria et al. (48)	Brazil	117,469 individuals	Intoxication, occupational exposure, and direct or indirect ingestion	Pesticides in general	Suicide attempts	Ecological/ Epidemiological	Pesticide poisoning increases the suicide rates	Worker's health and poisoning
Krawczyk et al. (49)	State of Alagoas	122,036 individuals	Intoxication, occupational exposure, and direct or indirect ingestion	Pesticides in tobacco production and those who used pesticides in any agricultural production.	Suicide attempts	Ecological/ Epidemiological	Combined effects between tobacco and pesticides may increase suicide attempt risk among agricultural workers	Worker's health and poisoning
Miranda Filho et al. (50)	Rio de Janeiro, Rio de Janeiro	710,000 inhabitants living in the studied area	Environmental	Pesticides in general	Death from CNS cancer	Database study	Increased rates of brain cancer mortality was observed in residents from rural areas	Cancer and SNC
Albuquerque et al. (51)	State of Pernambuco	990 individuals	Intoxication and occupational exposure	Pesticides in general	Pesticide poisoning	Database study	Rural workers were the main individuals that tried to suicide	Worker's health and poisoning
Machado and dos Santos (52)	Brazil	Brazilian population in the period (not clear)	Intoxication, direct or indirect ingestion	Pesticides in general	Suicide attempts	Database study	40% of the causes of deaths from self-intoxication related to pesticides	Intoxication
Motta et al. (53)	Botucatu, São Paulo	40 parturients and newborns	Environmental and parental exposure	Persistent organic pollutants	Pesticide contamination	Medical records	No correlations were reported.	Endocrine disruption and malformations
Ueker et al. (54)	Cuiabá, Mato Grosso	411 children under 5 years old	Environmental and occupational exposure	Pesticides in general	Presence of congenital malformations	Case-control	Association of paternal exposure to pesticides in agricultural-related work (OR = 4.65, 95% CI 1.03–20.98) and past paternal exposure to pesticides (OR = 4.15, 95% CI 1.24–13.66), associated with low maternal education (OR = 8.40, 95% CI 2).	Worker's health and malformations
Dutra and Ferreira (55)	Cascavel and Francisco Beltrão, Paraná	4,238 Newborns with congenital malformations	Environmental	Several	Birth defects	Ecological and population-based approach	High rates of congenital malformation were found in municipalities with high pesticide trade	Malformations
Buralli et al. (56)	Rio de Janeiro	82 farmers and families	Occupational exposure	Pesticides in general	Respiratory dysfuncitons	Interview	Pesticide exposure was significantly associated to spirometry impairment	Worker's health
Carmo et al., 2018. (57)	State of Bahia	858 individuals	Intoxication, direct or indirect ingestion	Pesticides in general	Suicide attempts	Database study	13.1% of the suicidal attempts were caused by pesticide and other chemical products ingestion.	Intoxication
	Nova Friburgo, Rio de Janeiro	352 individuals	Environmental and occupational exposure	Pesticides in general	Cancer risk in children	Interview	Reports association between exposure to pesticides in childhood and incidence of cancer and genetic polymorphism. Higher urinary concentrations of pesticides were found in children living in rural areas.	Worker's health and cancer
Oliveira et al. (58)	Campo Novo dos Parecis, Campo Verde, Diamantino, Lucas do Rio Verde, Nova Mutum, Pedra Preta, Primavera do Leste, and Sorriso, State of Mato Grosso.	1,081 newborns, 219 live births with congenital malformation and 862 healthy live births	Environmental, occupational, and parental exposure	Pesticides in general	Presence of congenital malformations	Population-based study	Maternal exposure to pesticides was associated with a higher occurrence of congenital malformations. Fetal malformations were statistically associated with male gender (OR = 1.50, 95% CI=1.11 – 2.04) and children of mothers who lived with a partner OR = 1.34 95% CI = 1.02 – 1.85. Positive associations were found between exposure to pesticides and malformation.	Worker's health and malformations
Gondim et al. (59)	Fortaleza, Ceará	410 cases	Intoxication, direct or indirect ingestion	Pesticides in general	Suicide attempts	Database study	30.2 of the cases were associated with pesticide ingestion.	Intoxication

## Results

### Pesticides Use in Brazil

**Figure 2:**
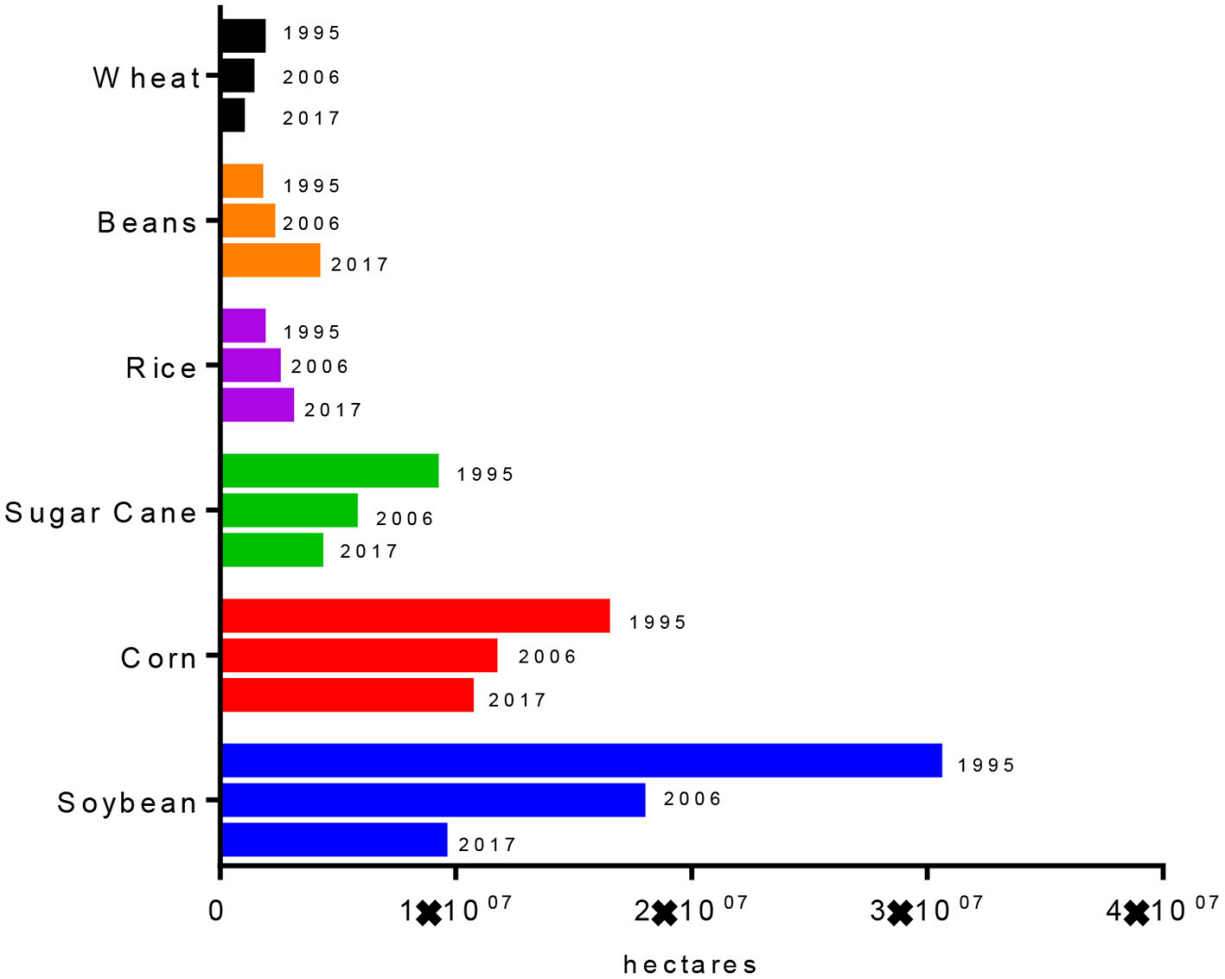
Harvested area of cultivated products from temporary crops-−1995, 2006, and 2017 according to records available at the Statistics and Geography Brazilian Institute (IBGE).

Agribusiness is regarded as one of the most important activities in the Brazilian economy. The grain production estimates of the Ministry of Agriculture, Livestock and Supply—MAPA (2020) point to a 2019/2020 harvest of 250.9 million tons, in a planted area of 65.5 million hectares. According to the most recent data from the Census of Agriculture, only three products are responsible for 85% of the area harvested from the products of annual crops, namely soybeans, corn and sugar cane (60). Also according to data from the Census of Agriculture, soybean monoculture occupied 30.4 million hectares in 2017; followed by corn monoculture, which covered 16.3 million hectares, and sugarcane, which occupied 9.1 million hectares. Soybean harvested area has more than tripled in Brazil over a period of ~20 years, rising from 9.4 million hectares in 1995 to 30.4 million in 2017 (Figure 2).

The high production of soybean, corn, and sugarcane is reflected in the prominent position that Brazil occupies both in the production and export of agricultural products. The country ranks first in exports of soybean and sugar, which markets its products to 43 destinations in the case of soybean, and 113 destinations in the case of sugar. Brazil is also the second largest corn exporter in the world, sending its production to 68 destinations (61). China and the United States are the main destinations for products from Brazilian agribusiness.

**Figure 3:**
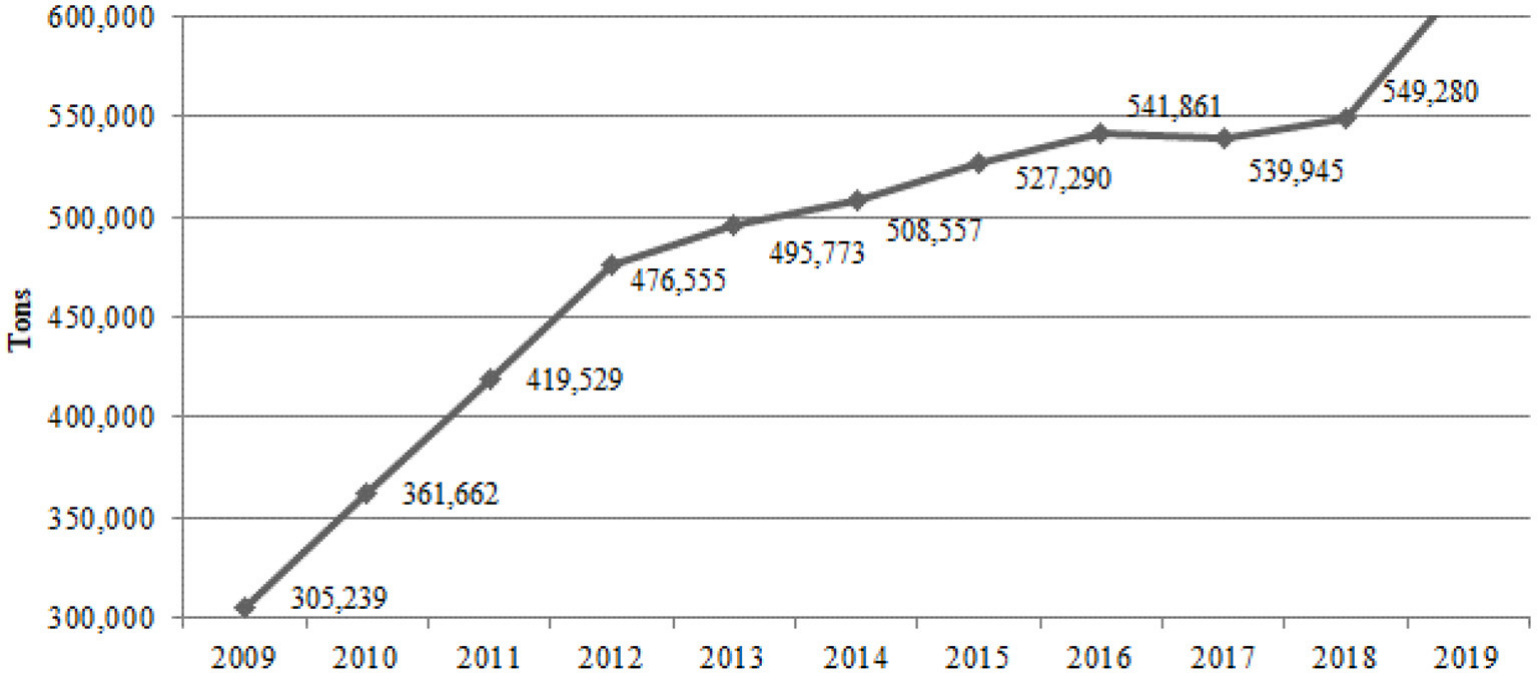
Tons of pesticides traded in Brazil from 2009 to 2019 according to IBAMA (2019).

Despite the significant exports and high profitability, the focus on the production of agricultural commodities and agrofuels brings the massive use of pesticides. Brazil appears on the international scene, together with the United States and China, as one of the main consumers of pesticides of the world (62). According to the Brazilian Institute of Environment (63), in 2014 the country surpassed the mark of 500 thousand of pesticides tons per year, and from 2009 to 2019 there was an increase of ~103% (Figure 3).

According to information from the National Union of the Industry of Products for Plant Defense (64), ~20% of the pesticides used in Brazil are of illegal origin. These fall into three categories and do not enter the official numbers presented in Figure 3: illegal smuggling, legal smuggling, and counterfeit products. In larger Brazilian regions there was an increased volume sold, such as in the North region (Amazon Region), which raised 504% from 2009 to 2019. According to agribusiness projections for the next decade, released by MAPA (2018), soybeans should show strong expansion in the northern states, especially Tocantins (34.8%), Rondônia (72.6%), and Pará (85.3%). In the other regions, the increase in the volume sold between 2009 and 2019 was also very significant: Midwest (196%), Northeast (175%), South (111%), and Southeast (91%).

**Figure 4:**
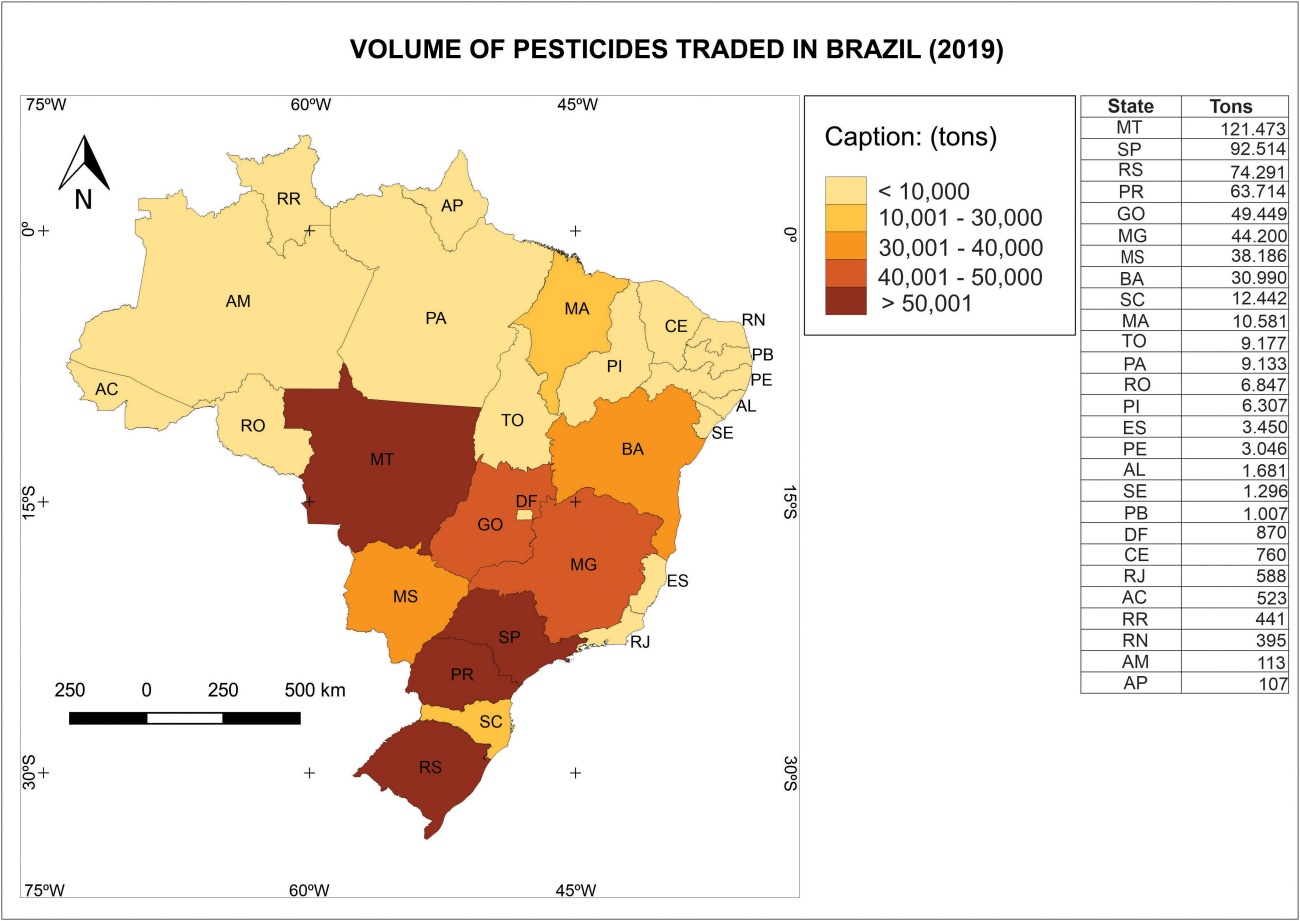
Volume of pesticides traded in Brazil during 2019 according to IBAMA (2019).

As shown in the map (Figure 4) the tons of pesticides traded by state in 2019. Mato Grosso (MT) was the biggest, reaching 121,473 tons, followed by São Paulo (SP): 92,514 tons; Rio Grande do Sul (RS): 74,291 tons; and Paraná (PR): 63,714 tons. In the second class are the states of Goiás (GO): 49,449 tons and Minas Gerais (MG): 44,200 tons, followed by the third class in which are the states of Mato Grosso do Sul (MS): 38,186 tons and Bahia (BA): 30,990 tons. In the fourth class of the map, Santa Catarina (SC): 12,442 tons and Maranhão (MA): 10,581 tons stand out. The other Brazilian states are in the class of those that used <10,000 tons of pesticides in 2019.

**Figure 5:**
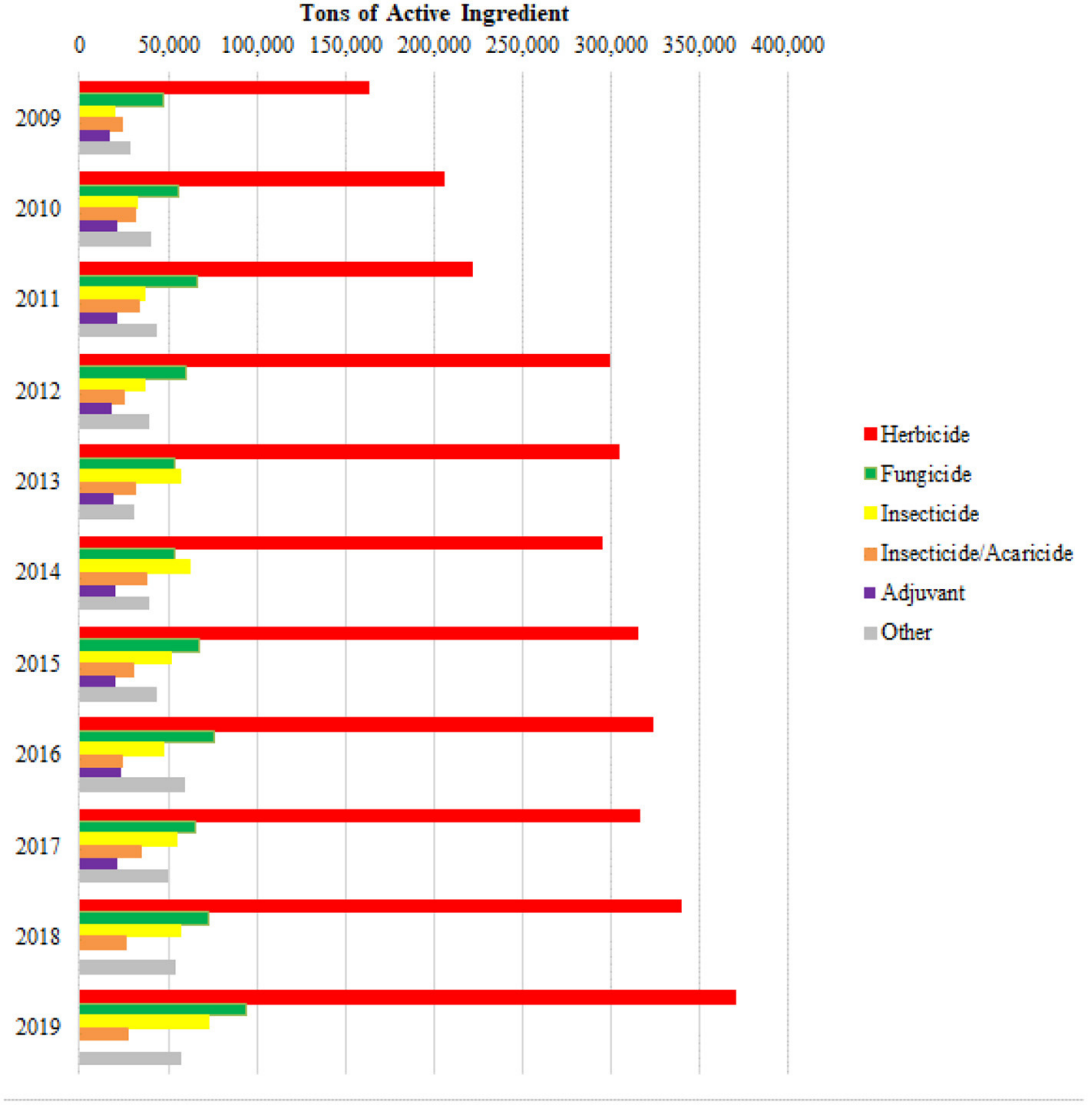
Marketing of pesticides according to their major chemical classes (2009–2019). There is no data on the sale of adjuvants for the years 2018 and 2019.

Pesticides include a large number of chemical molecules, with different modes of action and toxicity, being divided into three major classes: insecticides, fungicides, and herbicides (65). However, there are still acaricides, molluscicides, rodenticides, and among others. Herbicides are chemical substances that reduce or eliminate plants, popularly known as weeds, i.e., those that compete for water and nutrients with the cultivated plant. Currently, herbicides lead the list of the most commercialized active ingredients, with a growth of 126.5% between 2009 and 2019 in the sale of herbicides (63). Fungicides are chemical substances applied to cultivated plants to kill fungi or prevent the appearance of fungal diseases. Fungicide sales increased 100% from 2009 to 2019. Insecticides are products based on substances of direct or indirect action that cause the death of insects. Between 2009 and 2019, the greatest growth in the volume sold was among insecticides (259.6%). Insecticides/acaricides grew by 14.3%. The sales of adjuvants grew by 21.8% and that of other products, by 98.9% (see Figure 5).

Pesticides with herbicidal action that were most sold in Brazil in 2019 were Glyphosate and its salts, 2,4-D, Atrazine and Paraquat. This active ingredient had its commercialization prohibited in Brazil from September 22, 2020, due to toxicological reassessment as provided by Resolution RDC No. 177, of September 21, 2017, published in the Official Gazette of 22 September 2017. However, it can be used by producers who had stocked the product until August 31, 2021.Among the insecticides are Acephate, Melationa, and Chlorpyrifos. And, among the fungicides are Mancozebe and Chlorotalonil (63).

**Figure 6:**
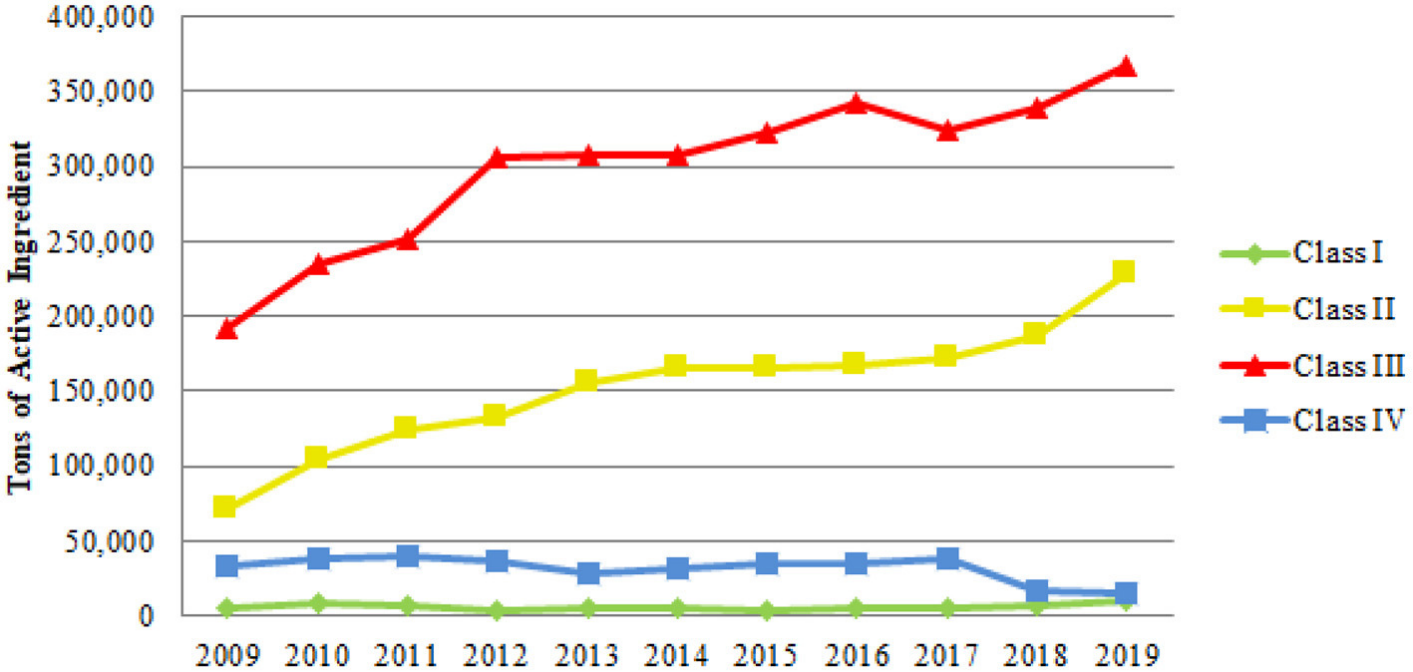
Tons of pesticides traded in Brazil concerning their environmental hazard risk (2009–2019).

As established in Decree 4,074/2002, IBAMA is the body responsible for “carrying out the environmental assessment of pesticides, their components and the like, establishing their classifications as to the potential for environmental hazard” (66). This assessment is based on the characteristics of the product, such as the physicochemical properties and their toxicity to organisms found in nature, how much the product accumulates in living tissues, whether it persists for a long time in the environment, if it can move in the soil, air or water, and the dangers of causing mutations, cancer, among other diseases, as well as the risk of compromising the reproduction of birds and mammals (67). The classes are divided as follows (Figure 6): Class I—Product Highly Dangerous to the Environment; Class II—Product Very Dangerous to the Environment; Class III—Product Dangerous to the Environment; and Class IV—Product Little Dangerous to the Environment. Thus, all pesticides registered in Brazil have one of these classifications of environmental hazard and this information is found in the product instructions. As shown in Figure 6, pesticides belonging to Class III are the most sold in the country. This fact occurs because the most commercialized active ingredients like Glyphosate and 2,4-D belong to Class III. Secondly, there are products classified in Class II, such as Atrazine, Mancozebe, Acephate, Chlorothalonil, and Malathione belong to this group. Thirdly comes Class IV products, and fourthly, Class I products. It is also noteworthy that a significant increase in the sale of pesticides classified as the most dangerous for the environment, and a reduction, in the last 2 years, of products belonging to Class IV, considered to be not very dangerous. Thus, there is a trend in the use of pesticides that are more harmful to soils and water resources, as well as to living beings that are exposed to these agents.

This shows a race against the world trend in favor of restrictions on the use of agrochemicals that pose a threat to environmental and human health, especially in countries of the European Union (68). In the next section, we will discuss the articulation of strategies by the government and companies in the agribusiness sector to make the use of pesticides in Brazil even more permissive.

### Impact of Pesticides on Human Health: Data About the Exposure of Brazilian Population

#### Exposure to Pesticides and Occurrence of Damage to the Central Nervous System

Three articles were found that addressed the possible effect of exposure to pesticides resulting in neurological pathologies. The researched outcomes were: malformation of the Central Nervous System (CNS) (47), delay in cognitive development in children and adolescents (46), and brain neoplasm in adults (50).

Miranda-Filho et al. (50) and Campos et al. (46) are epidemiological studies; disease incidence data were obtained from the Mortality Information System (SIM) provided by the Ministry of Health *via* DATASUS; information on pesticide consumption and population data, from the Brazilian Institute of Geography and Statistics (IBGE). Only Campos et al. (46) was a cohort study that evaluated environmental, socioeconomic, cognitive, and biomarkers of endocrine alterations and contamination by organochlorine pesticides (OC) variables.

Campos et al. (46) assessed cognitive performance, using the WISC-III scale, of children and adolescents living in an area contaminated with OC: the association with factors related to exposure (time and place of residence), levels of thyroid hormones and serum of OC pesticides; in children aged 6–16 years, coming from a region highly contaminated by OC pesticides, in Cidade dos Meninos, in the municipality of Duque de Caxias, in the State of Rio de Janeiro (RJ). Statistical associations were made by multivariate regression. The following correlations were verified: place of maternal residence and breastfeeding time were not associated with cognition, except for executive function; levels of hexachlorocyclohexane (HCH) were associated with a reduction in performance areas, resistance to distraction (RD), and speed of processing (VP); Gamma-HCH was associated with a reduction in RD and VP; and dichloro-diphenyl-trichloroethane (DDT) was reported to have the lowest score of VP.

Cremonese et al. (47) researched, among other things, the mortality rates from Congenital Malformations of the Nervous System (ICD 10 - Q00 to Q07) in children under 1 year old from urban and rural regions, and the association between pesticide consumption. Data were adjusted for statistical correlations, Spearman correlation coefficients (r) and mortality rate ratios (RR), stratified by gender and type of microregion (urban or rural). Significant positive correlations between per capita consumption of pesticides and mortality rates due to CNS and CVS defects were observed in rural microregions, but not found in urban microregions. According to the authors, overall, mortality rates for CNS and CVS malformations in rural microregions were significantly higher in each pesticide consumption quintile compared to the lowest quintile in the two study periods, with elevations ranging between 10 and 30%.

Miranda-Filho et al. (50) investigated the mortality rate due to brain malignancy (ICD 10 - C71) in adult individuals of both sexes, from populations in metropolitan and mountain regions. The article also provides data on the 50% increase in the total sales volume in the entire state of pesticides in the Serrana Region, marketed in 1996 (IBGE). The study indicated that brain cancer mortality rates were on average 40% higher, in all age groups, in the population of the Serrana Region of Rio de Janeiro. In addition, it was observed that there was a higher relative risk of mortality from CNS neoplasia for the rural population born after 1954; the index is four times higher for the highland population born between 1980 and 1989, compared to the highland cohort of 1945–1949.

Overall, all articles on this topic found evidence that increased exposure to pesticides is related to CNS damage. Together, the articles brought data from 35,751 people from the South and Southeast regions of Brazil, who had high levels of consumption and/or contamination by pesticides. Due to the heterogeneity of the found studies, comparative mathematical analyses were not performed.

#### Exposure to Pesticides and Occurrence of Cancer

Five of the found articles discussed the occurrence of cancer as a result of the exposure to pesticides, obtaining a significant sample of 12,311,993 Brazilians from different regions of the country. Each author adopted different methodological approaches to assess the risk to different types of neoplasms. This heterogeneity made it difficult to compare the data and the found studies. In two studies (43, 50) statistical analyses were obtained from data provided by public agencies; Mendonça et al. (19) and Ferreira et al. (15) carried out case-control studies, while Koifman et al. (18) brought primary data from an indigenous population, compared to epidemiological rates in the village and in other cities. Only Ferreira et al. (15) and Mendonça et al. (19) collected information on pesticide exposures; the others estimated from regional agricultural data. In two other studies (15, 43, 50), it was observed that there was evidence of the relationship between exposure to pesticides (or living in the environment) and the risk of developing cancer.

Study conducted by Koifman et al. (18) evaluated the exposure to environmental factors, extremely low frequency electromagnetic fields (EMF), pesticides, and heavy metals in the Parkatêjê indigenous community, living in a village in the Amazon. Data were obtained from direct magnetic field measurements, DDT and OC pesticide biomarker surveys in 89 blood samples and heavy metal samples in 14 indigenous hair samples. Cancer rates in the Parkatêjê population in 1992 were of three cases (a 22-year-old man with acute lymphoblastic leukemia, a 20-year-old woman with leiomyosarcoma, and a 55-year-old woman with cervical cancer). The authors compared the incidence of cancer in the 20–29 years old age group and those in the general population in the same age group in different Brazilian cities, does not show the incidence in the community^*^ calculated the expected incidence with incidence rates of Porto Alegre-RS, Belém-Pa, and Marabá-PA; and found that a reduction of 0.07 cases in this age group would be expected. The probability of this event occurring by chance was considered remote by the authors. Detected levels of EMF exposure were low (<1.0 mg) in 62% of measurements, and moderate levels (1.0–1.9 mg) were observed in 33% (seven people, all of them young adults). However, at the sites just below the transmission lines, which is close to the village, the EMF level reached 95 mg. Additionally, high levels of p,p'-DDT were observed in the studied samples, suggesting previous exposure to DDT and environmental contamination, since the studied group had no direct exposure to pesticides. Elevated levels of barium (Ba), magnesium (Mg), manganese (Mn), and strontium (Sr) were observed when compared to controls from the general population.

Regarding the occurrence of breast cancer, Mendonça et al. (19) investigated exposure to OC pesticides as a risk factor in 177 women diagnosed with invasive breast cancer and 350 control women selected from hospital visitors, all from the Metropolitan Region of Rio de Janeiro. Information was collected on possible pesticide exposures, menstrual history, reproductive history, family cancer, smoking, alcoholism, breast size, educational level, and anthropometric data. No statistically significant association was found between breast cancer risk and serum DDE level or history of pesticide exposure. In addition, the women studied were at greater risk for breast cancer when they had a family history, their breasts were large, or when they were alcohol consumers.

Curvo et al. (43) evaluated the childhood cancer morbidity and mortality associated with the agricultural use of pesticides in the State of Mato Grosso, Brazil. The group surveyed death rates from malignant neoplasms (ICD 10 - C00 to C97) in children and adolescents (up to 20 years old), from SIM, *via* DATASUS, and data on the use of pesticides in the same period were obtained with the Institute of Agricultural Defense of the State of Mato Grosso (INDEA-MT). The correlation between the average use of pesticides, new cases, and deaths from cancer was tested in the age group 0–19 years old, in the municipalities of Mato Grosso, in the periods between 2000 and 2006. Statistically significant results were found both for morbidity from cancer in children under 20 years old (*p* = 0.021), as well as for mortality from childhood cancer (*p* = 0.005), with a 95% confidence interval.

Ferreira et al. (15) investigated the association between the occurrence of leukemia in children under 2 years old with *in utero* exposure to pesticides; for that, a case-control study was carried out in 13 Brazilian states, from 1999 to 2007, in different cancer treatment centers. The survey included 252 children diagnosed with acute lymphoid leukemia (ALL) (*n* = 193) or acute myeloid leukemia (AML) (*n* = 59), and 423 children, from the same cities, who were in hospital care for non-diseases, neoplasms. Sociodemographic data of the children and information on exposure to pesticides in the preconception and breastfeeding periods were collected. The authors found that maternal pesticide exposures in the preconception period were significantly associated with ALL and AML in children aged 0–11 months old, and AML in children aged 12 to 23 months old. They also reported that maternal exposure to pyrethroids and unspecified solvents was associated with leukemia in children up to 2 years old, and that reported maternal exposure to permethrin increased risk estimates for AML in children up to 11 months old. Children whose mothers were exposed to pesticides 3 months before conception were at least twice as likely to be diagnosed with ALL in the 1st year of life when compared with those whose mothers did not report such exposure. Thus, maternal exposure to pesticides related to agricultural activities showed an increased risk for ALL and AML in children up to 2 years old.

#### Effects of Pesticides on Rural Worker's Health

The impact of exposure to pesticides on the health of Brazilian farmers and/or their families was addressed in 17 articles. Voluntary or accidental poisoning in rural workers, regardless of the outcome, was addressed in 13 articles (15, 20, 25–30, 33, 34, 48, 49, 51). Studies that addressed only rural residents were not included.

In nine studies, data were collected through questionnaires applied directly or indirectly to the workers (15, 20, 25–28, 33, 34). In only three, material was collected for biological analyzes of pesticide contamination (25, 33, 34). The questionnaires applied were directed to socioeconomic, environmental, health and comorbidity characteristics, forms and frequencies of exposure to pesticides (use of personal protective equipment, disposal of agrochemical packaging, and technical assistance), symptoms related to exposure to pesticides, and perception of the risks of using pesticides.

Four were epidemiological studies based on databases from government institutions. All studies found evidence that rural workers are at greater risk than the urban population of: voluntary and accidental pesticide poisoning, suicide and suicide attempts caused by pesticides (29, 30, 48, 49).

One article compared the data obtained in governmental notification systems from the Information of Notifiable Diseases (SINAN), Toxicological Assistance Center of Pernambuco (CEATOX), and SIM databases; and found disparities between “Place of occurrence of exposure” vs. “The exposure/contamination was due to work/occupation” (17% of cases), and “Report of Accident at Work (CAT)” vs. “The exposure/contamination was due to work /occupation?” in 25.96% of the cases, suggesting an under-reporting of accidents at work (51).

Other health problems found in farmers were: congenital abnormalities (39, 58), spontaneous abortions, infant mortality of up to 1 year of life (20). One study did not find evidence that maternal occupation in the agricultural sector was related to the higher risk of occurrence of orofacial clefts in newborns (24). Complaints related to the immune system (frequent fever, itchy skin, eyes, and nose), musculoskeletal system (joint pain), CNS and Peripheral Nervous System (PNS; dizziness, tingling in the upper limbs, sleep disorders, and vomiting) were also recorded (20), as well as respiratory symptoms (asthma symptoms, chronic respiratory diseases) (27) and pulmonary dysfunctions (56), and cognitive and neurobehavioral disorders, of the CNS and PNS (33).

Despite the few found studies, these show that damages to the health of rural workers and their families exist and are very varied, and can affect any organic system, including cognitive and mood alterations, even increasing the risk for various types of cancer. Even in this context, there are still no specific public health programs for this population. The articles also indicated under-reporting of occurrences of pesticide poisoning, especially in the category of exposure or contamination resulting from work or occupation. Exposure to pesticides among rural workers is heterogeneous, whether by contamination, quantity and variety of agrochemicals used, or age at exposure. This makes it difficult to characterize possible short- and long-term health impairments.

#### Malformations and Endocrine Changes

In the literature analyzed, it was observed that since 2003 to the present day, several authors have published studies on the investigation of exposure to pesticides and congenital malformations, showing a concern related to the effects that pesticides have on genetic, hereditary, and environmental levels, since Brazil has become one of the biggest consumers of pesticides (41). In addition, other selected works also showed a relationship between exposure to pesticides and reproductive and pregnancy disorders and clinical parameters of the newborn.

When the country is broken down into its states, the State of Mato Grosso stands out as a major agricultural producer and, consequently, the biggest national consumer of pesticides (58). Based on the above, in the same state, a case-control study was carried out with 219 live births with congenital malformations and 862 healthy live births, selected through the Live Birth Information System, between 2000 and 2009. From 2000 onwards, from the definition of the sample, the average use of pesticides in the months before and after fertilization was estimated, using bivariate statistical analysis and logistic regression, resulting in significant associations (*p* < 0.05) for congenital malformation, against exposure between the 3 months prior to fertilization and the first gestational trimester.

A case-control study with a sample of 174 cases and 274 controls performed by Ueker et al. (54) in Cuiabá, capital of the State of Mato Grosso, investigated the parents' previous exposure to pesticides and the occurrence of deformities in the offspring, resulting in higher maternal risks for mothers with a low level of education (OR = 8.40, 95% CI 2.17–32.52), father's work related to agriculture (OR = 4.65, 95% CI 1.03–20.98), and past paternal exposure to pesticides (OR = 4.15, 95% CI 1.24–13.66).

Likewise, an ecological study in the South and Southeast of Brazil analyzed the per capita consumption of pesticides and infant mortality rates, which concluded significant correlations between exposure and death due to CNS and cardiovascular alterations in children under 1 year old (14), also corroborating the case-control study by Silva et al. (39), with 42 cases of newborns with birth defects vs. 84 controls, in which it was statistically possible to identify differences between the groups and exposure to pesticides was shown to increase the risk of occurrence of birth defects when the parents acted or resided near crops or urban-rural borders.

According to Leite et al. (24), exposures can occur in various ways, namely, by residential proximity to rural and industrial areas, direct and unprotected contact with agricultural and domestic pesticides, and occupational and vector control exposure, increasing the potential risk factor for malformations, described in the case-control study (1:2) with 274 patients with orofacial clefts.

Relating to the article by Leite et al. (24), Dutra and Ferreira (55) reported on the exposure to these compounds and the relationship with the malformation, highlighting congenital alterations classified as cryptorchidism, malformations of the circulatory system (OR = 5.32, 95% CI = 4.48–6.31), and cleft lip and palate (OR = 1.62, 95% CI = 1.15–2.30). However, it reports non-summed fetal deaths and miscarriages, as being important biases.

The consumption of pesticides reported in another Brazilian state, the State of Paraná, considering the active ingredients available for a set of municipalities called Regional Units (URS). According to Dutra and Ferreira (55), 68 active ingredients were selected, from 262 obtained by the Agricultural Defense Agency of Paraná (Adapar), as they are eligible as endocrine disruptors, since, of the 20 URS, 11 had pesticide consumption above 01 ton, noting that 32.6% were flagged as endocrine disruptors, with the compounds glyphosate, atrazine, acephate, 2,4-D, and epoxiconazole/pyraclostrobin, exceeding 50% of the total of used pesticides. Endocrine disruptors or disruptors are substances that can interfere with the normal functions of the endocrine system, leading to problems with reproduction and development in both humans and wild animals (69).

Cremonese et al. (41), in an epidemiological survey with an ecological design, found the association between the per capita consumption of pesticides and adverse events during pregnancy in the southern region of Brazil, highlighted in the microregions with the highest consumption of pesticides. Pesticides showed the highest occurrence of premature births (<22 weeks) and unsatisfactory 1st and 5th min Apgar scores (<8) in both sexes.

On the other hand, Motta et al. (53) evaluated blood samples collected from 40 parturients and their newborns (NB) living in a rural area of Botucatu, in the State of São Paulo, and analyzed the index of contamination by pesticides and metals. However, there was no significant correlation (*p* > 0.05) between the maternal and newborn contamination index, as well as the newborn's perinatal clinical parameters, suggesting that the placenta can act not only as a barrier, but also as a filter, making the passage of toxic compounds difficult.

Another study looked for p,p'DDE in the bloodstream of a case-control group of infertile (*n* = 15) and fertile (*n* = 21) women, resulting in the presence of p,p'DDE in 100% of the women who sought treatment to become pregnant and 95.3% of pregnant women. They also observed that the levels of chemical compounds present in infertile women (case group) were higher than those detected in the control group (Mann-Whitney test: *U* = 56; *p*-value = 0.001) (42).

Koifman et al. (23) also analyzed the impact of pesticide exposure on human health. For this, the authors analyzed the volume of pesticide sales in 1985 and the reproductive changes described in the 1990's in 11 Brazilian states, showing through the Pearson correlation coefficient, a moderate-to-high correlation ranging from 0.36 to 0.81 mortality between 50 and 69 years old for breast cancer (*r* = 0.81, 0.41–0.95), ovarian cancer (*r* = 0.71, 0.19–0.92), prostate cancer, spermogram (*r* = 0.60, −00.1–0.88), and hospitalization rates for testicular cancer. Even though the study showed biases, such as the probability of the sale and consumption of the product not being in the same places, the results suggested that the reproductive disorders presented a decade later might be related to exposure to pesticides.

Regarding agrochemical residues in the human body, Delgado et al. (21), in research, analyzed the levels of persistent organochlorine pesticides: o,p'DDT, p,p'DDT, p,p'DDD, p,p'DDE, Aldrin, Dieldrin, Endrin, Heptachlor, Heptachlor-epoxide, alpha, beta, and gamma-Hexachlorocyclohexane, Hexachlorobenzene, and polychlorinated biphenyls. In blood samples from 33 volunteers living in the urban area of the city of Rio de Janeiro, it was possible to detect p,p'DDE in 51.5% of the samples, with concentrations ranging from 1.4 to 8.4 μg/L of serum, or, based on fat, from 0.200 to 3.452 μg/g of serum lipid. However, the median levels of p,p'DDE were much higher in the older age group (76.9%; 0.401 μg/g lipids), with no significant difference between the sexes at older ages (men: 71.4%, 0.407 μg/g lipids and 83.3% women, 0.378 μg/g lipids).

Similar results of exposure to pesticides from blood samples of 42 individuals from a total of 102 individuals aged 6–16 years old, studied in the city of Meninos, State of Rio de Janeiro, in which several pesticides were identified in the serum, highlighting the presence of alpha-, beta-, and gamma HCH, p,p'DDE, and p,p'DDT in over 90% of individuals. However, the aim of the study was to assess the level of cognitive performance of this population in an area contaminated with OC pesticides, in which an association between chronic exposure and the mother's place of residence was shown, what could lead to cognitive deficits in these children and adolescents.

#### Intoxications

Of the 47 articles selected for evaluation of the full text, 25 articles that discussed intoxications, both intentional and unintentional were identified. The predominant regions were those with extensive use of pesticides, especially the southeast of Brazil, mainly the State of Rio de Janeiro, with nine published studies (21, 45), followed by the State of Pernambuco (3) (20, 48, 51), Minas Gerais (2) (25, 34), Mato Grosso do Sul (2) (25, 29), Paraná (2) (15, 57), São Paulo (1), Alagoas (1) (49), Bahia (1) (57), and Fortaleza (1) (59). A study of the cities in the Serra Gaúcha region (20), as well as two studies (48, 52) that did not specify the region, analyzed data from Brazil using information systems. The population studied comprised 9,971 individuals, with variation between groups: children (5, 108) (17, 31, 36, 38), adolescents (25) (40), rural workers (1,590) (20, 27, 28), adults (2,740) (15, 25, 26, 33, 51), and search through reports of the rural population (475) (29). Regarding the population of the studies that report on notifications of intentional poisonings and suicides in adults, a total of 242,338 cases (30, 34, 48, 49, 59) is comprised. Of these, suicide attempts (666) (30, 32) and completed suicides (240,962) (30, 34, 48, 49, 57, 59), of which 85,828 cases were in people over 60 years, were registered. The most researched types of pesticides were pesticides (6) (35, 40, 49, 52, 57, 57), organochlorines (4) (17, 21, 25, 29), carbamate-based pesticides (4) (20, 25, 30, 34), insecticides (4) (26–28, 38), fungicides (8), “chumbinho” (3) (32, 45, 59), and household cleaning products, such as disinfectants and bleach (41). One article did not mention the type of pesticide it studied (48).

Most studies show that poisoning caused symptoms, especially acutely, such as headache, nausea, decreased vision, dizziness, skin irritation, loss of appetite, tremors, vomiting, allergic crisis, and diarrhea (20, 25–28, 48, 49, 51). Asthma and chronic respiratory disease, as well as late neuropathy, were also reported as health problems due to chronic contact with pesticides (15, 27, 33, 34, 48, 49, 52, 59). However, a single study (33) reported that inhabitants of the urban area of the city of Rio de Janeiro had a low body load of persistent organochlorines.

Practically 80% (20) of those handling pesticides were unaware about personal protective equipment (PPE), 92% reported not using any type of PPE either in the preparation or in the application of pesticides. Ninety-eight percent wash their hands after use (26) and report dizziness, nausea, and headache; seven of them had health problems due to exposure to pesticides, four required medical care and had been hospitalized. The empty packages of the products were buried in the ground (37%), stored for later burning (18.5%), or had no previously established destination (44.5%); most packages (54.4%) were left in the field or had another inappropriate destination, such as the common garbage dump. Yet, 52% of the respondents mentioned the reuse of packaging for domestic use (26). When asked about the use of pesticides on a daily basis in the field, 13.2% reported that they had already suffered some type of poisoning, most needed medical help; 45 out of 159 reported feeling unwell during product application (20, 25, 48, 49, 51).

According to the logistic regression model, the rural worker who does not use personal protective equipment (PPE) has up to 72% chance of becoming intoxicated and workers who have the seller as their advisor in the purchase and use of pesticides are 73% more likely to become intoxicated (25); 62% of the respondents report not having received technical assistance for the protection or application of pesticides (26). The chance of intoxication for individuals who mentioned at least one organophosphate or carbamate as the main pesticide is 115% higher compared to those who did not mention any product from this chemical group (25). As for the symptoms of chronic respiratory diseases, there was a risk increase of 70–90% among individuals who worked on more than one farm, prepared chemical mixtures, and washed contaminated clothes when compared to those who did not carry out these activities (27). Only one study reports the relationship between the interviewees' understanding of the environment and their perception of environmental risks, such as river contamination (28). In Cidade dos Meninos, in the municipality of Duque de Caxias, in the lowlands of the State of Rio de Janeiro, exposures to Dichlorodiphenyltrichloroethane (DDT) and Dioxin exceed the minimum acute, intermediate, and chronic risk levels for soil and food, such as eggs and milk (35). Excessive risk of cancer for children and adults due to exposure was also found (35).

When the approach was about the domestic use of chemical products and poisoning of children under 6 years old, of the total of 1,574 occurrences, 39% was of poisoning by household chemicals and 15% by some type of pesticide (38). In this interim of poisoning, cases of suicide attempts and consummated acts stand out, of which 21 of the 25 cases of female adolescents between 13 and 19 years old were caused by spontaneous forms of poisoning by carbon monoxide or pesticides (40). The use of a type of rodenticide known as “chumbinho” was reported by over 20% of the respondents (52, 59) and 70% of the records of completed suicides were caused by the use of medications such as psychotropic drugs (1). Among the age group analyzed, the average suicide rate was 6.4 cases, with higher suicide rates among people aged 35–64 years old and among men aged 15–34 years old (48). In a comparative study of suicide cases among agricultural and non-agricultural workers, 122,036 deaths were analyzed, of which 15,671 cases were of agricultural workers and 106,365 were of non-agricultural workers (49). However, there was an increasing trend in the mortality rate by suicide in the elderly population through self-intoxication by pesticides and chemicals, totaling 858 deaths in the period from 1996 to 2013 (28).

## Discussion

The studies included in this review showed that exposure to pesticides has a relevant impact on the health of the Brazilian population, regardless of age and gender, and on workers in rural areas or not. Most poisoning events seem to result from the continuous use of pesticides, whether occupationally or environmentally, characterizing a public health problem. The ways in which these compounds are known have a direct impact on their use; evidence of the impact of pesticides on both public health and the environment are many and widely reported in the literature. Thus, we make some considerations below in order to contextualize the results of these studies.

It is estimated that each Brazilian consumes an average of seven liters of pesticides per year, as it is directly related to the 70,000 acute and chronic poisonings in our country, according to data from a dossier prepared by the Brazilian Association of Public Health (ABRASCO) (8). The Brazilian Ministry of Health warns that, for each notified pesticide-poisoning event, there are another 50 not reported (41). Other studies are in line with what we identified in this study, regarding unintentional poisoning affecting children in greater proportion (BRASIL). The national statistics of poisoning in children shows a predominance (58.5%) of the accidental cause, followed by suicide attempts by adolescents. For every three accidental cases, there is an attempt or a suicide by young people (BRASIL; MS; FIOCRUZ). In adults, the rate of attempts increases significantly, of which over 60% are due to the use of psychotropic medications (53), given that this is 10% lower than what our study found.

As for the data presented at the Brazilian level regarding intentional poisonings by the Notifiable Diseases Information System (SINAN), there were over 29 thousand pesticide poisonings confirmed in the last decade. And just as in this study, regarding poisoning attempts, “chumbinho” was also used, a product that has been banned in the country since 2012. In a study carried out in Taiwan between 2011 and 2019, there was a marked reduction in suicide rates by pesticides after the ban of Paraquat in 2018, compared to the expected suicide rates based on the linear pre-ban trends of 2011–2017 (53), which is not verified in this study, that shows the use of pesticides considered prohibited in the country in suicide attempts.

Among the pesticides most reported in this study, the most harmful to the human body are found, such as insecticides, herbicides, and rodenticides. Among them are the organochlorines, banned in Brazil since 1985 because they are responsible for leaving permanent residues in the fatty tissues of mammals, birds, and fish and can remain in the environment for over 100 years (70). The toxicity of these compounds has been confirmed in many studies, especially with animals (71, 72), finding that there is the induction of enzymatic activities by free radicals, culminating in altering the immune response, lipid metabolism, and vitamin transport and, consequently, affecting the reproductive processes. Data report that these compounds are considered mutagenic, teratogenic, and carcinogenic (73).

Thus, as mentioned in this study, other authors showed that both rural workers and populations living close to the plantations suffer the consequences of the use of pesticides above the recommended rates (74–76), contributing to such damages to occur. However, a single study in this research reports that urban inhabitants of the Metropolitan Region of Rio de Janeiro have a low body load of persistent organochlorines. Nevertheless, the damage caused by pesticides is not limited only to individuals who are exposed to the environment, but to the entire chain of individuals who live there. Some species do not die when coming into contact with pesticides, whether by ingestion or other means, but accumulate these toxics in their bodies, passing them through the food chain, thus harming other species (71, 72), making the need for more explicit studies that relate this exposure to possible harm to the planet as a whole, as a single study in this review reports the relationship between respondents' understanding of the environment and the perception of environmental risks, such as river contamination (28).

A study carried out with blood analysis of pest control workers in Colombia reinforces that when they report their occupational activity, it is directly linked to the worker's acute and chronic poisoning, which presented equivalent data, ranging from neurological symptoms such as: headache (28.7%), dizziness (29.9%), gastrointestinal symptoms such as: diarrhea (9.2%), vomiting (2.3%), culminating in neuropsychiatric symptoms (24%), which may trigger suicide attempts (77). Regarding the knowledge of farmers about the use of Personal Protective Equipment (PPE), it is necessary to raise awareness among the applicators, because although the products applied are regulated, they pose risks to human health. In an interview, the agribusiness analyst of the Federation of Agriculture and Livestock of the State of Minas Gerais (FAEMG) reports that acute poisoning due to pesticides tripled in the region in the year 2019 (59). This is consistent with our data when we report that 80% (27) of the pesticide applicators were unaware of PPE, 92% reported not using any type of PPE either in the mixture's preparation or in the application of pesticides.

Some authors also report cases of poisoning of rural workers for not using protective equipment, washing the equipment in a domestic tank, and using a manual backpack sprayer. In these cases, the risks of intoxication can increase from 16 to 535% (78, 79).

Based on our study, we can infer that, due to the lack of awareness and knowledge of individuals, the practice of inappropriate disposal of pesticide containers is still common, even though there is legislation that specifically addresses this issue, such as the pesticide law (Law 7,802, of July 11, 1989) and its regulations (Decree No. 98,816, of January 11, 1990). Among the articles surveyed, the empty packaging of the products was buried in the earth (37%), stored for later burning (18.5%), or did not have a previously established destination (44.5%); most of the packaging (54, 4%) was left in the field or had another inappropriate destination, such as the common garbage dump. And yet, 52% of the respondents mentioned reusing packaging for domestic use (6), which corroborates data researched at the Brazilian level, demonstrating the seriousness and danger in relation to health that encompasses the theme of awareness in the field (55, 80).

Besides all these factors related to health problems resulting from poisoning, the studies presented here also show the importance of the studies carried out to assess the effects of occupational and environmental exposures in relation to congenital malformations, reproductive disorders, and pregnancy. The methodologies used were varied, including observational studies, with data collection, and experimental, with a dosage of pesticides in the blood and evaluation of clinical parameters.

Several studies carried out in other countries are in line with the findings of the studies carried out in Brazil that were presented here. Jaacks et al. (81) demonstrated that the dosage of pesticides in the urine of pregnant women in the city of Bangladesh was significantly correlated with higher rates of preterm birth, low birth weight, and shorter length for gestational age. Likewise, Parvez et al. (82), showed similar results when they measured the pesticide glyphosate in the urine of pregnant women living in the city of Indiana, United States, and observed that 93% of women had levels above the detectable limit of the pesticide and that this parameter was correlated with shorter duration of gestation time. Hu et al. (83) measured pesticide metabolites in the urine of 615 Chinese women who wanted to become pregnant and followed them for 1 year. In the end, they concluded that higher levels of organophosphates and pyrethroids were related to higher rates of infertility.

The main limitation of our study is the difficulty to establish a quality assessment concerning the selected studies. Several studies had an observational design or were based on a secondary source of data, limiting our access to some of the parameters that compose the quality score (study design, sample size, exposure assessment, measurement of effect, and/or control of confounding factors). This may be because most of these studies did not intend to evaluate occupational exposures, and were based on occasional exposures by people who lived in pesticide-contaminated areas or high pesticide trade regions, which impaired a more precise analysis.

In conclusion, this study provided an opportunity for a deeper understanding of the harm and impacts that pesticides can cause on the lives of beings and that awareness and the correct dissemination of information directly impact practices regarding the current and prospective community scenario throughout the Brazilian region.

## Data Availability Statement

The original contributions presented in the study are included in the article/supplementary material, further inquiries can be directed to the corresponding author.

## Author Contributions

All authors listed have made a substantial, direct, and intellectual contribution to the work and approved it for publication.

## Conflict of Interest

The authors declare that the research was conducted in the absence of any commercial or financial relationships that could be construed as a potential conflict of interest.

## Publisher's Note

All claims expressed in this article are solely those of the authors and do not necessarily represent those of their affiliated organizations, or those of the publisher, the editors and the reviewers. Any product that may be evaluated in this article, or claim that may be made by its manufacturer, is not guaranteed or endorsed by the publisher.
